# Motor sequence learning occurs despite disrupted visual and proprioceptive feedback

**DOI:** 10.1186/1744-9081-4-32

**Published:** 2008-07-25

**Authors:** Eric D Vidoni, Lara A Boyd

**Affiliations:** 1 Department of Physical Therapy & Rehabilitation Science, University of Kansas Medical Center Kansas City, KS, USA; 2 School of Rehabilitation Sciences, University of British Columbia Vancouver, British Columbia, Canada

## Abstract

**Background:**

Recent work has demonstrated the importance of proprioception for the development of internal representations of the forces encountered during a task. Evidence also exists for a significant role for proprioception in the execution of sequential movements. However, little work has explored the role of proprioceptive sensation during the learning of continuous movement sequences. Here, we report that the repeated segment of a continuous tracking task can be learned despite peripherally altered arm proprioception and severely restricted visual feedback regarding motor output.

**Methods:**

Healthy adults practiced a continuous tracking task over 2 days. Half of the participants experienced vibration that altered proprioception of shoulder flexion/extension of the active tracking arm (experimental condition) and half experienced vibration of the passive resting arm (control condition). Visual feedback was restricted for all participants. Retention testing was conducted on a separate day to assess motor learning.

**Results:**

Regardless of vibration condition, participants learned the repeated segment demonstrated by significant improvements in accuracy for tracking repeated as compared to random continuous movement sequences.

**Conclusion:**

These results suggest that with practice, participants were able to use residual afferent information to overcome initial interference of tracking ability related to altered proprioception and restricted visual feedback to learn a continuous motor sequence. Motor learning occurred despite an initial interference of tracking noted during acquisition practice.

## Background

Motor learning requires the ability to adjust future performance based on information regarding prior execution. The feedback that is necessary for this process can come from exogenous sources such as coaching, or endogenous sources such as self-evaluation of performance and afferent feedback, including vision and proprioception.[1] Recently, vision and proprioception have received considerable attention in the literature regarding their role during motor learning.[2] The findings suggest that the two modalities support learning of different environmental characteristics. For example, it appears that only vision is necessary for adapting to new kinematic environments such as a visuomotor shift.[2] In contrast, learning to control novel dynamics such as manipulating a new object can be acquired through the proprioceptive system alone.[3] In particular, previous work has demonstrated the importance of proprioception in adapting movement coordination to external forces encountered by the limb during movement.[4,5]

Our understanding of the importance of proprioception for motor sequence learning is limited by two factors. First, past work investigating the interactions among proprioception, vision and motor learning has typically employed discrete movements [5-7] that participants are already familiar with and thus, have an established, well-learned motor plan.[6] Second, research has not carefully dissociated permanent changes in behavior that represent motor learning from short-term performance changes [8] by employing follow-up, retention testing session.[2,7,9] Commonly, participants have been allowed to familiarize themselves with (i.e., practice) the task prior to data collection.[2,5,10] Though this creates a controlled environment for investigating the role of proprioception in short-term motor performance, it confounds our understanding of motor learning by essentially pre-training participants on the task. This issue was inadvertently highlighted by Bevan and colleagues [10] who anecdotally reported that participants demonstrated the best performance for the tasks that they initially practiced. Rather than examining adaptation of familiar movements in novel environments, in the present research we sought to examine motor sequence learning of an entirely new motor pattern under conditions that altered visual and proprioceptive feedback.

Little prior work has focused on the importance of proprioception during continuous motor sequence learning. This omission is surprising considering that proprioception has been suggested as an integral component of feedback-based skill learning.[11,12] During motor learning, proprioceptive feedback may form a template for comparison to a motor plan; perhaps through the tuning of electromyographic activity via feedback-based adaptation.[11] Extending these findings, Hwang and Shadmehr noted that computer simulations of muscle spindle-based learning closely matched human learning of a reaching task in a force field.[12]

We addressed previous experimental gaps by asking participants to practice a novel, continuous motor pattern over the course of 2 days and return on a separate day for retention testing to assess motor learning. We severely restricted visual feedback and introduced vibration to alter proprioception. Vibration has previously been used to alter proprioception during upper extremity movement. Vibration can predictably shift perception of movement as demonstrated by Goodwin and colleagues [13] who cataloged its illusory effects on sensory perception. This effect has been repeated and further qualified [4,5,14-18] making vibration a useful experimental tool for the study of motor control and learning.[19] The application of vibration to the arm has been used in past experiments to determine the role of proprioception in the execution of previously learned movements [17,18] and the control of the limb against external forces.[4,5] Vibration produces the illusion of movement by stimulating primary spindle fiber afferents.[14] Furthermore, vibration can mask the report of accurate afferent information regarding antagonist muscle stretch via stimulating primary spindle fiber afferents [15] and influencing muscle activation patterns.[16] In light of these findings, in Experiment 1, we characterized the impact of vibration at the shoulder on proprioception using a limb position matching task. We confirmed that application of vibration at the shoulder predictably altered proprioception and caused participants to misjudge motion in the limb-matching task.

After confirming that our method of vibration alters proprioception, we used the same method to apply vibration during practice of a continuous tracking task and examined the effect of altered limb proprioceptive sensation on motor sequence learning. We hypothesized that if proprioception was essential for motor sequence learning, altering this feedback during practice would be evident in accuracy measures at retention testing. Alternately, it is possible that the normal motor system is flexible and robust enough to facilitate sequence learning even when proprioception is altered. The possibility that individuals can demonstrate motor sequence learning even when proprioception is altered is important to neuroscientists and clinicians alike. Knowledge of the significance of accurate proprioceptive information during motor sequence learning may facilitate the genesis of novel models that predict the capacity skill acquisition.

### Experiment 1

#### Methods

To confirm the putative effects of vibration in advance of our investigation of motor learning (Experiment 2), we first verified the effect of vibration on both passive and active continuous, whole-arm movement patterns. Previous studies have applied shoulder muscle vibration during whole arm movements to examine motor performance.[18] Because our experimental task required shoulder flexion and extension movements to push and pull a frictionless lever mounted at shoulder height, application of vibration to the proximal arm musculature was ideal. We expected vibration to predictably alter perception of arm movement during limb matching in Experiment 1.

#### Participants and Apparatus

**Figure 1:**
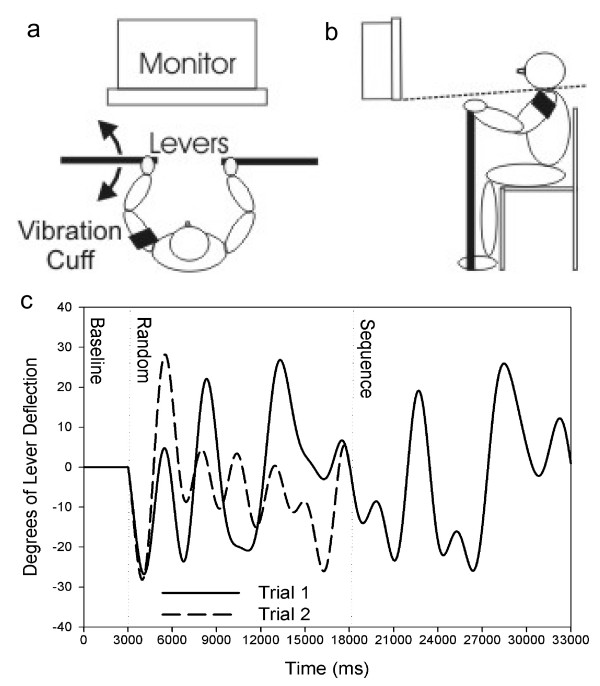
**Experimental Setup**. a) Participants were seated before a computer monitor and gripped one (Experiment 2) or both (Experiment 1) horizontally mounted levers. A vibrating cuff was secured to one arm. b) Draping was drawn over the shoulders to prevent visualization of arm movement, represented by a dashed line. c) In Experiment 2, participants followed a pattern of movement similar these two example trials. Following a 3s stable baseline, sine-cosine waveforms dictated target movement. Two trial waveform patterns, each assembled from 1 random and 1 repeating sequence, are overlaid. The random epoch comes first, followed by the repeated sequence epoch during both trials for ease of visualization.

Fifteen healthy adults (8 males, 7 females; mean age 33.1 [range 26–46]) with no reported diabetes or upper extremity sensorimotor impairment participated. Each provided informed consent in the manner prescribed by the University of Kansas Medical Center Humans Subjects Committee, in compliance with the Helsinki Declaration. Participants engaged in a previously reported limb position matching task modified for the upper extremity.[20] The protocol required the participant to continuously estimate the movement of a passively displaced extremity while experiencing vibration to one of their arms. We modified and extended the protocol to include vibration of both active and passive upper extremities. Two nearly frictionless, horizontally mounted levers were positioned at shoulder level to allow participants to grasp one in each hand (Figures 1a and 1b). The levers were attached to potentiometers that registered angular displacement.

Three eccentrically loaded motors within a cuff provided the vibratory stimulus. The cuff was secured to the dominant arm, as identified by the Edinburgh Handedness Inventory,[21] with an elastic wrap at the level of the deltoid insertion. The vibrating motors were positioned on the anterior, lateral and posterior aspects of the upper arm. In this manner aspects of the biceps brachii, triceps brachii and deltoids were vibrated.

Vibration-induced movement illusion occurs over a wide range of stimulation frequencies, but most effectively at 50–100 Hz.[15,22] To avoid the possibility that participants would accommodate to vibration, thus potentially limiting its impact on limb proprioceptive sensation, we varied the frequencies of vibration (50, 60, 70, 80 Hz) across trials; for Experiment 1 two trials were performed at each frequency in a quasi-randomized order. The same 16, 30s random movement patterns were tested for each condition. These patterns were similar in design to those seen in Figure 1c, without a repeated component. Following the waveform creation protocol of Wulf and Schmidt,[23] these waveforms were balanced across the midline with regard to amplitude, meaning that flexion and extension movements of equal magnitude were required for each trial.

#### Experimental Conditions

To confirm that our method of vibration altered proprioceptive sensation, we tested limb position matching during both passive and active movement, with and without vibration. Eight trials were performed under each condition. The participant's eyes were closed throughout each trial. In all trials one passive arm was driven through a continuous movement pattern by an experimenter while the participant matched that movement with the opposite, active arm. Vibration was introduced in half the trials. For example, a right-handed participant would be fitted with the vibration cuff on the right upper arm. In the passive, driven condition the examiner would guide the right arm and the individual would match those movements with the left arm for 8 trials without vibration and 8 trials with vibration. Arm guidance was accomplished by the experimenter supporting arm weight at the elbow and moving the lever. Care was taken to minimize experimenter-participant contact. In the active, matching condition the examiner would guide the left arm and the individual would attempt to match with the right (Table 1). Thus we were able to compare typical limb position matching ability to that when vibration was applied either to the active, matching limb or to the passive, driven limb. To avoid potential vibration aftereffects, non-vibration trials were always performed prior to vibration trials.[24]

**Table 1:** Limb Position Matching Protocol

	Active, Matching Vibraton Condition		Passive, Driven Vibraton Condition	
	Left	Right	Left	Right
Side Vibrated		X		X
Active Matching Arm		X	X	
Passive Driven Arm	X			X

Test conditions for a right-handed subject in the limb position matching task, Experiment 1. The vibrating cuff was applied to the dominant upper limb (in this case, the right shoulder). Then, each arm was moved, or "driven", by the experimenter, while the participant actively matched those movements with the opposite arm. In this manner, the right arm was vibrated both while it was actively matching and while it was passively driven.

#### Outcome Measure

The displacement of each lever and thus the movement of each arm was sampled at 40 Hz, raw position data were smoothed using a 100 ms moving average, and data from each arm was corrected for constant error. To demonstrate and quantify the effect of our vibratory manipulation, the perceptual shift stimulated by vibration was indexed by the ratio of the active, matching and passive, driven arm movement amplitudes (Eq. 1).

(1) RMS ratio = RMS position of active tracking arm/RMS position of driven arm Root mean square (RMS) = SQRT(∑xi^2^/n) where xi = limb position

RMS ratios over 1.0 indicate movement amplitude of the active, matching arm was greater than that of the driven arm. RMS ratios during no-vibration trials were averaged for each participant. Additionally, correlation coefficients were calculated to assess spatiotemporal coordination between arms. Based on previous work,[13,20] we predicted that vibration delivered to the passive, driven arm would result in the perception of movement amplitude in that arm to be greater than in reality. In that experimental condition, individuals would overestimate the magnitude of active limb motion required to match the position of the passive, driven arm. The opposite was expected when vibration was applied to the active, matching arm.

One-way ANOVA was used to compare active, matching arm vibration, passive, driven arm vibration and no-vibration conditions. Post-hoc comparison of vibration and no-vibration conditions was planned using one-tailed Student's t-test to further explore expected condition differences, α = .025.

## Results

**Figure 2:**
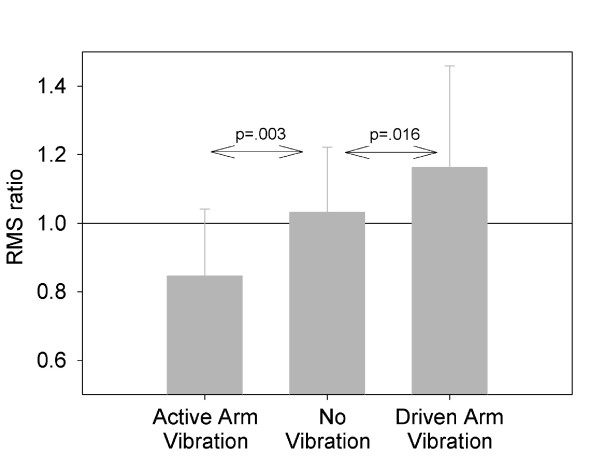
**Effect of Vibration on Limb Excursion Sense**. During the limb position matching task, participants interpreted the vibrated arm as having moved to a greater extent than in reality. This resulted in significantly reduced (active matching arm vibration) or increased (passive driven arm vibration) ratio of RMSE measures when vibration was applied as compared to when vibration was not applied.

We hypothesized that if our method of vibration resulted in altered proprioceptive sensation, then regardless of which arm was vibrated (active tracking or passive driven) participants would interpret the vibrated arm as experiencing greater excursion than in reality. As can be seen in Figure 2, vibration to the shoulder musculature resulted in changes in perceived upper limb movement consistent with our expectations. Our condition ANOVA revealed statistically significant differences between conditions, (F(2,42) = 6.997, p = 0.002). The average RMS ratio was significantly different on non-vibration trials than on the passive driven arm vibration trials (p = 0.016) and active vibration (p = 0.003) conditions. Limb movement between sides was closely related across conditions (mean r = 0.93 ± 0.07). Anecdotally, some participants reported feeling vibration both proximally in the neck as well as distally in the elbow and "tingling" into the wrist and hand.

## Conclusion

These data demonstrate that vibration to the upper extremity at frequencies between 50 and 80 Hz resulted in altered proprioceptive sensation that did not accurately reflect the true state of the limb. Importantly, every 0.1 difference in the RMS ratio (Figure 2) translates into an average 3 cm difference between hand positions at the transition between flexion and extension movements. As our participants demonstrated vibration-induced RMS ratio differences of 0.19 in the active, matching arm vibration condition and 0.13 in the passive, driven arm vibration condition vibration substantially altered tracking accuracy.

Based on our findings, and other recent work by Bock et al. [19] outlining the problematic and invasive nature of other methods of sensory disruption (i.e., ischemic cuffs, peripheral nerve blocks) we elected to use the same method of vibration in Experiment 2 to disrupt proprioception throughout practice of a continuous motor tracking task. This approach allowed us to ascertain the impact of peripherally altered proprioception on motor sequence learning.

### Experiment 2

In Experiment 1 we found that our method of applying vibration to the upper arm alters proprioceptive signaling and results in a misperception of limb state. In Experiment 2 we sought to capitalize on this effect to examine the impact of altered proprioceptive sensation during motor skill practice on learning of a continuous motor sequence.

## Methods

### Participants

Twenty-five healthy adults (9 males, 16 females; mean age 27.0 [range 22–43]) with no reported diabetes, or upper extremity muscular or sensory impairments agreed to participate. Each provided informed consent in the manner prescribed by the University of Kansas Medical Center Humans Subjects Committee, in compliance with the Helsinki Declaration. Three of these individuals also participated in Experiment 1. The dominant arm, as determined by the Edinburgh Handedness Inventory [21] was used for the task.

### Tracking Task

Seated in front of a computer monitor, participants used their dominant arm to track a continuously moving target that followed a sine-cosine wave pattern [23,25]: 23 right-handed, 2 left-handed. The same lever set-up used in Experiment 1 (Figures 1a and 1b) was moved with shoulder flexion and extension to track an on-screen cursor vertically up the screen (shoulder flexion) or down the screen (shoulder extension). Naturally, elbow extension followed shoulder flexion and elbow flexion accompanied shoulder extension in a parasaggital plane. The target appeared as a white box and participant's movements were represented as a yellow circle cursor. The lever apparatus necessitated 31 cm of angular excursion over a maximum of 60°, to accurately track the waveform; each participant was easily able to move through this range of motion. As in Experiment 1, lever displacement sampling was performed at 40 Hz. All stimuli were presented at 40 Hz using custom software developed on the LabView platform (v. 7.1; National Instruments, Austin, TX).

The pattern of target movement was predefined according to a method modified from Wulf and Schmidt.[23] For each 33s trial, a unique target wave was assembled from one 3s baseline, presented at the middle of the screen and tracking range (to allow participants to orient their arm to task midline), and two 15s component sine-cosine wave segments, or "epochs" (Figure 1c). In each tracking trial, participants were exposed to a novel random waveform epoch and an epoch that contained a repeated waveform sequence. To avoid order effects, presentation of the repeated sequence epoch randomly occurred as the first or second segment with a trial. The same presentation order of trials was employed for every participant.

### Experimental Design

Upon enrollment, individuals were randomly assigned to either have their passive, non-tracking arm vibrated as an experimental control condition (CTL), or to have their hand-dominant active, tracking arm vibrated (AV). Prior to starting, participants were instructed to track the target as accurately as possible by controlling the position cursor with shoulder flexion/extension movements of the lever.

Individuals practiced the experimental tracking task 50 trials a day for two days (Table 2). During these training days, vibration was applied according to group assignment. The possibility of accommodation to the vibratory stimuli was avoided by randomly varying the frequency of stimulation after each trial; frequencies of 50, 60, 70 and 80 Hz were randomly arranged and then delivered in the same order for all participants.

**Table 2:** Testing Schedule and Conditions

**Day**	Blocks	Trials	Vibration	Visual Feedback
Training Day 1	1–2	20	Yes	Faded
	3–5	30		200 ms/2s
Training Day 2	6–10	50	Yes	200 ms/2s
Retention Day 3	11	10	No	200 ms/2s
	12	10	Yes	200ms/2s

Each subject completed three (3) days of training and retention testing. Visual and proprioceptive conditions followed this schedule. Participant groups (CTL, AV) differed only according to the side on which vibration was applied.

On a separate third day, participants returned for retention test trials. We wished to examine the impact of proprioception on motor sequence learning over the two previous training days without the transient effects of altered proprioception influencing performance. Therefore, during the first block of retention testing the cuff was fitted according to group assignment but no vibration was introduced. To confirm the effect of vibration on motor performance as compared to motor learning, after the retention test was completed *without *vibration, participants performed an additional block with vibration applied according to their group assignment (CTL = vibration to the passive, non-tracking arm; AV = vibration to the active, tracking arm).

The possibility that participants rely on vision to compensate for altered proprioception was accounted for by 1) preventing participants from seeing either of their arms, and 2) severely restricting visual feedback of the cursor position. For both groups draping was used to prevent vision of the arms throughout the entire study. The drape was placed over, but did not come in contact with, the participant's upper body to avoid the possibility that brushing against it would provide cutaneous sensory cueing. Additionally, over the first 20 practice trials, visual feedback regarding lever position was faded (i.e., linearly reduced). We determined that initially (early practice) some visual feedback of cursor position was necessary for participants to understand the task; however, this feedback was removed quickly (our schedule of fading was based on that reported by Winstein and colleagues).[26]

Past work investigating continuous sequence production demonstrated that when visual feedback for cursor movements was delivered at 500 ms/1 sec or less it actually disrupted the use of visual feedback to guide movement.[27] Thus, in instances where visual feedback is less than or equal to 500 ms/1 sec, Kao showed that its brevity rendered it virtually useless for guiding hand-controlled cursor movements. Applying this finding, we linearly reduced the amount of time the position cursor appeared beyond the threshold reported by Kao. This ensured that visual error-feedback could not be used to continuously guide movement. In the present task, arm position information was faded from continuous delivery on trial 1 (block 1), to a 200 ms position cursor presentation every 2s by trial 19 (block 2) and kept at this level for the remainder of the study. To maintain motivation for this difficult task and encourage accurate tracking, participants were provided summary feedback after each trial during acquisition as a percentage of time the position cursor spent within a 10° bandwith of the target. Because summary feedback regarding overall tracking accuracy was provided only for motivational purposes, did not contain sufficient information to alter performance, and was not explicitly manipulated across the groups we also provided this information at retention.

### Outcome Measures

The primary outcome measure was root-mean-squared error of velocity changes (RMSE) (Eq. 2) separately calculated for the random and repeated sequence epochs as the area difference between target and participant movement velocity.

(2) RMSE = SQRT(∑(xi - Xi)^2^/n) where xi = probe velocity, Xi = target velocity

RMSE from each 10 consecutive trials was averaged to represent 1 block of sequence performance during the initial two days of training, and learning at retention. We considered the pattern of continuous velocity changes rather than absolute position [23,28,29] as recent work has emphasized the encoding of velocity-based information by the proprioceptive system. [7,30]

### Statistical Analyses

First, to ensure that no baseline motor control or epoch-related differences biased performance, two-way ANOVA of Group (AV, CTL) and Epoch (random, sequence) at Block 1 with repeated measures correction for Epoch was performed on RMSE. Next, change in tracking RMSE over the 2 practice days was assessed via three-way ANOVA of Group × Epoch × Block (1–10) with repeated measures correction for Epoch and Block. Sequence-specific learning was assessed using the retention test with two-way ANOVA of Group (AV, CTL) × Epoch with repeated measures correction of Epoch using both position and velocity error data. During practice and at retention, our analysis of random versus sequence epochs allowed us to parse out improved motor control or non-specific learning from more permanent changes in behavior as a result of sequence-specific learning for the repeated epoch.[23,25,28] Finally, we assessed the cost of vibration to sequence-specific performance by calculating the difference in RMSE between random and sequence epochs when vibration was again introduced on day 3. One-way ANOVA of Group was used to test for change in performance. All analyses were tested at α = .01 to protect against Type I error. A Greenhouse-Geisser correction was used where appropriate.

Box-plot analysis revealed that one individual in the AV group performed poorly enough to be statistically considered an outlier when compared to the group during every block of practice. This participant was excluded from further analysis.

## Results

### Continuous Tracking During Acquisition Performance

At the beginning of training in block 1, before visual feedback of the position cursor was faded, performance for the AV and CTL groups was similar. No main effects of Group or Epoch or interaction of the two were noted (p > 0.05).

**Figure 3:**
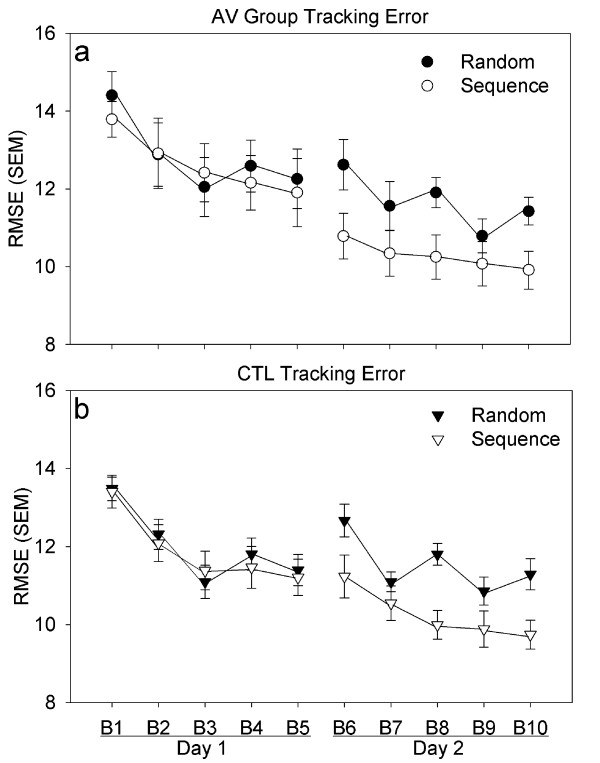
**Tracking Error**. Average RMSE over skill practice (days 1 and 2). Open shapes represent performance on the sequence epoch, closed shapes represent the random epoch. Panel (a) displays the performance of the AV group that experienced vibration to the active tracking arm. Panel (b) displays the performance of the CTL group that experienced vibration to the passive, unused arm. Block 1 represents initial performance with attenuating visual feedback but also vibration. The remaining blocks with vibration and minimal visual feedback, show an interaction of epoch and block suggesting improvement on sequence epoch tracking over time. Decreased RMSE, towards graph bottom, denotes performance improvements.

Visual inspection of the data shows that over the course of the two training days all participants improved (decreased) sequence tracking error as compared to random tracking performance (Figure 3). This was confirmed via a three-way ANOVA; the acquisition of sequence-specific knowledge across practice is evident in the significant Epoch × Block interaction (F(9,198) = 24.211, p < .001). However, no Group effect or interaction was evident (p > 0.1).

### Continuous Tracking at Retention

**Figure 4:**
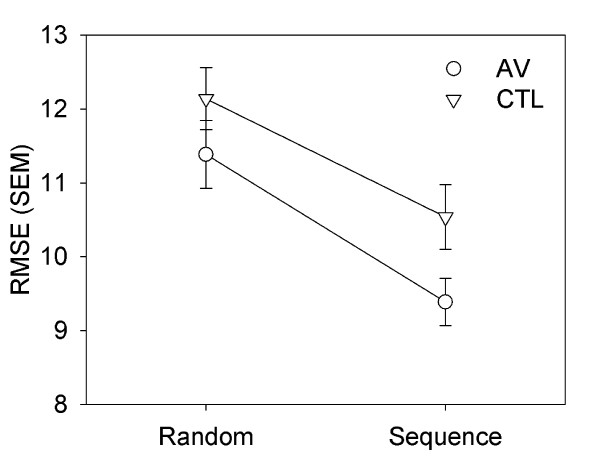
**Sequence-specific Learning**. At retention, when vibration was removed but visual feedback continued to be disrupted, improvements on sequence epoch tracking persisted regardless of group, showing that altered proprioception during acquisiton performance did not impair continuous motor sequence learning. Decreased RMSE, towards graph bottom, denotes performance improvements.

At the day 3 retention test without vibration both groups demonstrated sequence-specific learning of task regularities that allowed them to maintain improved tracking ability for the sequence epoch when compared to random performance (Figure 4). This was confirmed with a two factor ANOVA Group × Epoch where only the main effect of Epoch reached significance (F(1,22) = 37.407, p < .001).

**Figure 5:**
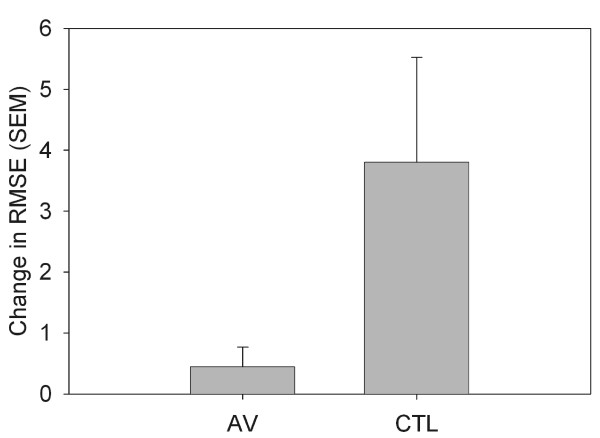
**Reintroduction of Vibration**. On day 3, during the last block of the study, sequence-specific learning improvements in tracking that were demonstrated by the AV group at the retention test when vibration was removed were masked by the re-introduction of vibration. That is, performance on random and sequence epochs were similar as shown by the lack of difference (low change score) between random and repeated sequence tracking error for the AV group. In contrast, CTL participants maintained sequence-specific improvement that was seen in no-vibration retention testing. Larger change in RMSE denotes greater performance difference between the sequence than the random epoch.

When vibration was reapplied according to group assignment on the retention test day, we noted a cost to expression of sequence-specific learning for the AV group but not the CTL group. That is, vibration resulted in a meaningful (Effect Size = 0.74) decline in tracking accuracy for the repeated sequence. The interfering effect of vibration was evident in the loss of learning-related difference between tracking error for repeated as compared to random epochs for the AV but not the CTL group (Figure 5). This effect trended toward significance (F(1,22) = 3.667, p = 0.069).

## Conclusion

The purpose of this study was to examine the impact of altered proprioception and reduced visual feedback on continuous motor sequence learning. A large body of literature demonstrates that information regarding body state is crucial for motor control.[4,6,31-33] In this study, we sought to determine whether this was also the case for motor sequence learning. In the past, Rothwell and colleagues [34] suggested that motor learning might be deleteriously impacted by absent proprioception via their case report of a deafferented individual. In this case, the authors reported that learning new, complex sequences of hand movements was difficult when deafferentation was present. We wondered if similar negative effects would be present for continuous motor sequence learning when proprioception was shifted by vibration. We discovered that in the short-term, peripherally altered proprioception and reduced visual feedback impacted motor performance; however, given two days of practice, the extraordinarily robust human motor learning system was able to overcome the challenge presented by shifted proprioceptive sensation and motor learning of a repeated continuous sequence occurred.

To our knowledge the present study represents the first experimental investigation of the impact of altered proprioception on continuous motor sequence learning. The experimental design employed for the current work differs from previous studies in several important ways. First, we used a continuous tracking task that required participants to use their entire upper extremity to produce movement. It has been suggested that investigation of complex movements (i.e. those that involve more degrees of freedom or greater muscle activation) are critical for understanding motor learning and behavior.[35,36] Our task met those criteria by including multiple joints, greater movement excursion and longer movement patterns. Also, previous studies of proprioception have often employed discrete, reaching-type tasks for which participants likely already have at least a rudimentary motor plan.[2,3,6,7] Our use of an entirely novel continuous tracking task allowed us to more fully examine novel motor sequence learning. Finally, we engaged individuals in two days of practice and a separate, delayed, retention test. In this manner learning versus performance improvements were clearly differentiated.[8] Because no prior studies of the role of proprioception have employed a retention test design, it has not been clear whether altered proprioception would deleteriously impact motor learning.[2,7]

We hypothesized that if veridical proprioceptive sensation was essential for sequence learning, peripherally altered proprioceptive information that did not reflect the true state of the limb would diminish both acquisition and retention of the repeated motor sequence. We discovered that the opposite was true; all participants were able to learn sequence-specific regularities as compared to random epoch performance. The finding that individuals can learn to accurately and continuously track a repeating sequence even when vibration was applied to the arm being used suggests that accurate and intact proprioception are not absolute prerequisites for encoding and consolidating movement regularities. We found that the group that practiced with altered proprioception (AV group) and minimal visual feedback was able to improve in the same manner as the group who experience only control vibration to the non-tracking limb. Additionally, we found that when vibration was re-introduced at retention, motor learning in the AV group was masked by altered performance; this effect was not observed for the CTL group. These findings were facilitated by our experimental design; had we stopped data collection after 1 day as past work has done we would not have noted the positive effect of task practice in overcoming altered proprioceptive feedback.

Cordo and colleagues [33,37] have suggested that the dynamic position and velocity information supplied by proprioceptors may be important for the execution of movement sequences. Based on this, we posited that proprioception would also be critical for learning the spatio-temporal regularities of a repeated continuous sequence. Rather, we found that accurate proprioceptive information was not essential for learning our experimental task. Nor was continuous visual feedback. Recent work has reported that motor sequence learning can occur in a range of other environmental experiences. Overduin and colleagues [38] demonstrated that sequence learning occurred independently of learning predictable shifts in the dynamic environmental state. Our work supports and extends these findings to show that motor sequence learning can occur despite changes in visual and peripheral proprioceptive information.

Simply becoming aware of the repeating sequence is one possible reason that the AV group was able to learn the continuous tracking task. It is certainly possible that observation of target movement was sufficient to stimulate learning. Indeed, sequence learning has been demonstrated following stimulus observation alone [39] especially when individuals attend to the task.[40,41] In accordance with these findings we cannot rule out the possibility that untrustworthy proprioception was compensated for by paying greater attention to target motion.

Another plausible explanation for our finding that altered proprioception did not diminish learning may be that accurate afferent sensation from more distal segments of the arm might have been preserved and exploited. We cannot totally rule out this possibility with the present experimental setup. Single joint elbow muscles as well as wrist and finger musculature, joint and cutaneous afferents were possibly spared from vibratory disruption (though several subjects reported "numbness and tingling" into the forearm and wrist). Furthermore, secondary spindle afferents appear to be relatively insensitive to vibration.[15] The central nervous system could have preferentially attended to these signals for information regarding performance.

However, we suggest that the hypothesis outlined above cannot completely explain our results because this same "unaltered" afferent information did not overcome vibration-induced changes as shown by the limb position matching task in Experiment 1 or during reintroduction of vibration at Experiment 2 retention testing. These findings supply convergent evidence that vibration was disruptive to motor control. Based on these findings, it appears that vibration induced at least some shift in the afferent feedback from the shoulder and elbow spanning musculature to the central nervous system that altered motor output. Motor sequence learning appears to have occurred despite this shift in the veracity of limb proprioceptive sensation.

It has been previously noted that vision is critical when proprioceptive sensation is diminished or absent[34,42] Ghez et al. [31] reported that individuals with large fiber sensory neuropathy improved their aim on discrete reaching tasks when able to visualize arm position before movement. To explore the contribution of proprioception without the confound of visual feedback, we reduced visual information available to the participant via several controls. First, we occluded vision of the arm via draping. Next, we quickly faded feedback regarding cursor position over the first 20 trials to an intermittency exceeding that which Kao [27] cited as being disruptive to continuous tracking. However, we chose to preserve some visual feedback to reduce cumulative error which might have obscured improved motor control associated with learning [43] by displaying the arm position cursor for 200 ms at 1800 ms intervals. It is possible that even this minimal visual information may have allowed participants to evaluate their performance and adjust accordingly in the absence of trustworthy proprioceptive feedback. However, based on the past work of Kao [27] we find this explanation of our conclusions highly improbable.

Our finding of preserved continuous sequence learning despite restricted visual feedback and altered proprioception reflects the dynamic and robust nature of a motor learning system that is able to compensate for inaccurate afferent information through redundant physiological and cognitive systems. One or some combination of all of the mechanisms proposed above may have facilitated learning for participants in this research. Though these findings do not directly support our original hypotheses that altered proprioception would disrupt motor sequence learning, they are not without precedent. Skill learning has been reported in dorsal rhizotomized monkeys.[44,45] The juxtaposition between our findings and Taub et al.'s are in contrast to reports by others,[46,47] who have reported disruption of skill learning following sensoricortical damage. These seemingly contradictory results may be a function of the difference between central and peripheral neural damage/disruptions. It remains to be seen if those with chronic sensory impairment resulting from damage to central sensory cortical or thalamic regions have difficulty learning new motor skills. Future work should consider this possibility in persons with medical conditions characterized by reduced proprioception.

## Competing interests

Neither author has any personal or financial relationship to declare regarding this manuscript.

## Authors' contributions

EDV participated in all aspects of the study. LAB participated in study design, interpretation of findings and drafting the manuscript. Both authors have read and approved the final version of this manuscript.
